# Neutrophil–lymphocyte ratio is associated with worse outcomes in patients with cirrhosis: insights from the PRO-LIVER Registry

**DOI:** 10.1007/s11739-025-03955-x

**Published:** 2025-05-20

**Authors:** Tania D’Amico, Marzia Miglionico, Roberto Cangemi, Giulio Francesco Romiti, Benedetta De Fabrizio, Salvatore Fasano, Fabrizio Recchia, Lucia Stefanini, Valeria Raparelli, Francesco Violi, Stefania Basili, P. R. O.-LIVER Collaborators, Giuseppe Palasciano, Felicia D’Alitto, Vincenzo Ostilio Palmieri, Daniela Santovito, Dario Di Michele, Giuseppe Croce, David Sacerdoti, Silvia Brocco, Silvano Fasolato, Lara Cecchetto, Giancarlo Bombonato, Michele Bertoni, Tea Restuccia, Paola Andreozzi, Maria Livia Liguori, Francesco Perticone, Benedetto Caroleo, Maria Perticone, Orietta Staltari, Roberto Manfredini, Alfredo De Giorgi, Maurizio Averna, Antonina Giammanco, Alessandro Granito, Irene Pettinari, Sara Marinelli, Luigi Bolondi, Lorenzo Falsetti, Aldo Salvi, Emanuele Durante-Mangoni, Flavio Cesaro, Vincenza Farinaro, Enrico Ragone, Ignazio Morana, Angelo Andriulli, Antonio Ippolito, Angelo Iacobellis, Grazia Niro, Antonio Merla, Giovanni Raimondo, Sergio Maimone, Irene Cacciola, Doriana Varvara, Davide Drenaggi, Silvia Staffolani, Antonio Picardi, Umberto Vespasiani-Gentilucci, Giovanni Galati, Paolo Gallo, Giovanni Davì, Cosima Schiavone, Francesca Santilli, Claudio Tana, Anna Licata, Maurizio Soresi, Giovanni Battista Bianchi, Isabella Carderi, Antonio Pinto, Antonino Tuttolomondo, Giovanni Ferrari, Paolo Gresele, Tiziana Fierro, Olivia Morelli, Giacomo Laffi, Roberto Giulio Romanelli, Umberto Arena, Cristina Stasi, Antonio Gasbarrini, Matteo Gargovich, Maria Assunta Zocco, Laura Riccardi, Maria Elena Ainora, William Capeci, Giuseppe Pio Martino, Lorenzo Nobili, Maurizio Cavallo, Pierluigi Frugiuele, Antonio Greco, Antonello Pietrangelo, Paolo Ventura, Chiara Cuoghi, Matteo Marcacci, Gaetano Serviddio, Gianluigi Vendemiale, Rosanna Villani, Ruggiero Gargano, Gianpaolo Vidili, Valentina Di Cesare, Maristella Masala, Giuseppe Delitala, Pietro Invernizzi, Giovanni Di Minno, Antonella Tufano, Francesco Purrello, Graziella Privitera, Alessandra Forgione, Valentina Curigliano, Marco Senzolo, Kryssia Isabel Rodríguez-Castro, Gianluigi Giannelli, Carla Serra, Sergio Neri, Mario Rizzetto, Wilma Debernardi Venon, Gianluca Svegliati Baroni, Gaetano Bergamaschi, Michela Masotti, Filippo Costanzo, Gino Roberto Corazza, Stephen Hugh Caldwell, Francesco Angelico, Maria Del Ben, Laura Napoleone, Licia Polimeni, Marco Proietti, Valeria Raparelli, Giulio Francesco Romiti, Eleonora Ruscio, Andrea Severoni, Giovanni Talerico, Filippo Toriello, Annarita Vestri, Lucia Stefanini, Lucas Rumbolà, Giovanni Buoninfante, Francesca Maiorca, Annamaria Sabetta, Simone Di Cola

**Affiliations:** 1 Department of Translational and Precision Medicine, Sapienza-University of Rome, Viale del Policlinico 155, 00161 Rome, Italy; 2 Department of Translational Medicine, University of Ferrara, Ferrara, Italy; 3 University Center for Studies on Gender Medicine, University of Ferrara, Ferrara, Italy; 4 Faculty of Nursing, Medicine, and School of Public Health Sciences, University of Alberta, Edmonton, AB Canada; 5 Department of Clinical Internal, Anesthesiological and Cardiovascular Sciences, Sapienza - University of Rome, Rome, Italy

**Keywords:** Cirrhosis, Inflammation, Neutrophil–lymphocyte ratio, Mortality

## Abstract

**Background:**

Liver cirrhosis (LC) is a leading global cause of morbidity and mortality, with inflammation playing a key role in disease progression and clinical complications of LC. The Neutrophil/Lymphocyte Ratio (NLR), a readily available marker of systemic inflammation, has been linked to short-term adverse outcomes in LC, but data on long-term follow-up are limited. This study aimed to investigate the relationship between NLR and long-term all-cause mortality in an unselected cohort of LC patients.

**Methods:**

Data were gathered from the Italian multicenter observational study “PRO-LIVER”. Patients with available data to calculate NLR at baseline were included. Baseline clinical determinants of NLR and the association of NRL with all-cause mortality at 2-year follow-up were evaluated.

**Results:**

From the overall cohort (*n* = 753), 506 patients with LC (31% female, mean age 64.8 ± 11.9 years) were included in the analysis. Median value of NLR was 2.42 (Interquartile Range [IQR]: 1.61–3.52). At baseline, patients with NLR ≥ 2.42 were more likely to have Child–Pugh B or C, hepatocellular carcinoma (HCC), or portal vein thrombosis (PVT). After a median follow-up of 21 months, 129 patients died: 44 (17%) with NLR < 2.42 and 85 (34%) with NLR ≥ 2.42 (*p* < 0.001). At multiple-adjusted Cox regression analysis, NLR ≥ 2.42 was independently associated with all-cause mortality (HR: 1.65; 95% CI: 1.12–2.44; *p* = 0.012), along with age, Child–Pugh C class, HCC and PVT.

**Conclusions:**

NLR is associated with long-term all-cause mortality in LC. NLR may serve as a potentially easily available tool to aid risk refinement in LC.

**Trial registration number:**

ClinicalTrials.gov Identifier: NCT01470547.

## Introduction

Liver cirrhosis (LC) is one of the most common causes of morbidity and mortality worldwide, and among the most widespread non-communicable diseases, with epidemiological changing trends in incidence, prevalence and underlying etiology [1]. Among the predisposing factors to LC, a state of inflammation has been extensively described, from the onset to the progression to the end-stage disease [2]. In this context, the inflammatory processes exert a significant influence, particularly through the dysregulation of the balance between activation and homeostasis of the immune system. Both innate and acquired immunity are involved in all stages of liver damage and regeneration, highlighting the complex interplay of immune responses in liver disease [3].

The neutrophil–lymphocyte ratio (NLR), defined as the ratio of the absolute value of neutrophils and lymphocytes in a peripheral venous blood stream, has been previously described as a non-invasive marker of the balance between the innate and adaptive immune response: neutrophils and lymphocytes are indeed indicators, respectively, of an active inflammatory state and of the regulatory pathway's activity in the immune system [4]. Prior studies demonstrated that NLR is an easily available, cheap and widespread biomarker of systemic inflammation as well as a valid prognostic marker in multiple conditions, including cardiovascular, infectious, and chronic inflammatory diseases [5].

In patients with LC, the clinical role of NLR as predictor of adverse outcomes has been hypothesized [6]. Indeed, previous studies in patients with advanced LC have shown an association between NLR and the higher risk of infection, acute decompensation, portal vein thrombosis, and both in short- and long-term mortality [7, 8]. Notwithstanding this prior evidence, there is still uncertainty on the potential applications of NLR in real-world practice, the severity of the disease in which this biomarker may play a prognostic role, as well as the optimal reference value to help the risk stratification of individuals with LC.

To fill this knowledge gap, we analyzed data from a prospective multicenter study specifically aimed to identify whether the NLR is associated with adverse outcome in a long-term follow-up among contemporary nationwide cohort of patients with LC at different severity stages.

## Methods

The PRO-LIVER (Portal vein thrombosis Relevance On Liver cirrhosis: Italian Venous thrombotic Events Registry; ClinicalTrials.gov Identifier: NCT01470547) study was a multicenter prospective study involving 43 centers in Italy (*n* = 33 internal medicine units, *n* = 10 hepatology centers), coordinated by the Italian Society of Internal Medicine (SIMI). The primary objective of the PRO-LIVER was to estimate the prevalence and incidence of portal vein thrombosis (PVT) in a cohort of patients with LC of any etiology and severity, with or without hepatocellular carcinoma (HCC). The presence of concomitant extra-hepatic neoplasms was the only exclusion criteria applied to the population. Full details on the inclusion and exclusion criteria, the procedures followed throughout the study, and the primary results of the study are reported elsewhere [9, 10].

Briefly, all patients enrolled in the study were followed-up for 2 years, with outpatient visits conducted every 6 months. Additionally, an annual re-evaluation was scheduled through the performance of instrumental control exams. Baseline characteristics, including demographics, comorbidities, and laboratory values, and all relevant clinical events occurring during the follow-up period were recorded and reported using a specific case report form, standardized for all centers involved in the study.

In this analysis, we included patients who had available data on neutrophil and lymphocyte count and stratified them according to their NLR levels. We also analyzed the association of baseline NLR with the incidence of major adverse outcomes along the 2-year follow-up. For this analysis, our primary outcome was defined as the occurrence of all-cause mortality.

### Statistical analysis

Categorical variables were reported as count and percentages, and differences were analyzed using chi-square test; continuous variable were reported as mean and standard deviation (SD) or median and interquartile range (IQR), according to whether these were normally or non-normally distributed; significant deviation from normality distribution was assessed visually. Comparisons of normally distributed variables were performed using Student’s *t* test, while non-normally distributed variables were compared using Mann–Whitney *U*.

Multiple-adjusted logistic regression model was performed to identify baseline clinical characteristics associated with higher levels of NLR at baseline.

Kaplan–Meier curves were reported, to evaluate the survival of patients with LC based on the median values of NLR. Differences in survival distribution between groups were analyzed using Log-Rank test.

A multiple adjusted regression Cox regression analysis was performed to identify variables independently associated with survival. A *p* value < 0.05 was considered statistically significant. All analyses were performed using SPSS v. 28 software (IBM, NY, USA).

## Results

**Fig. 1:**
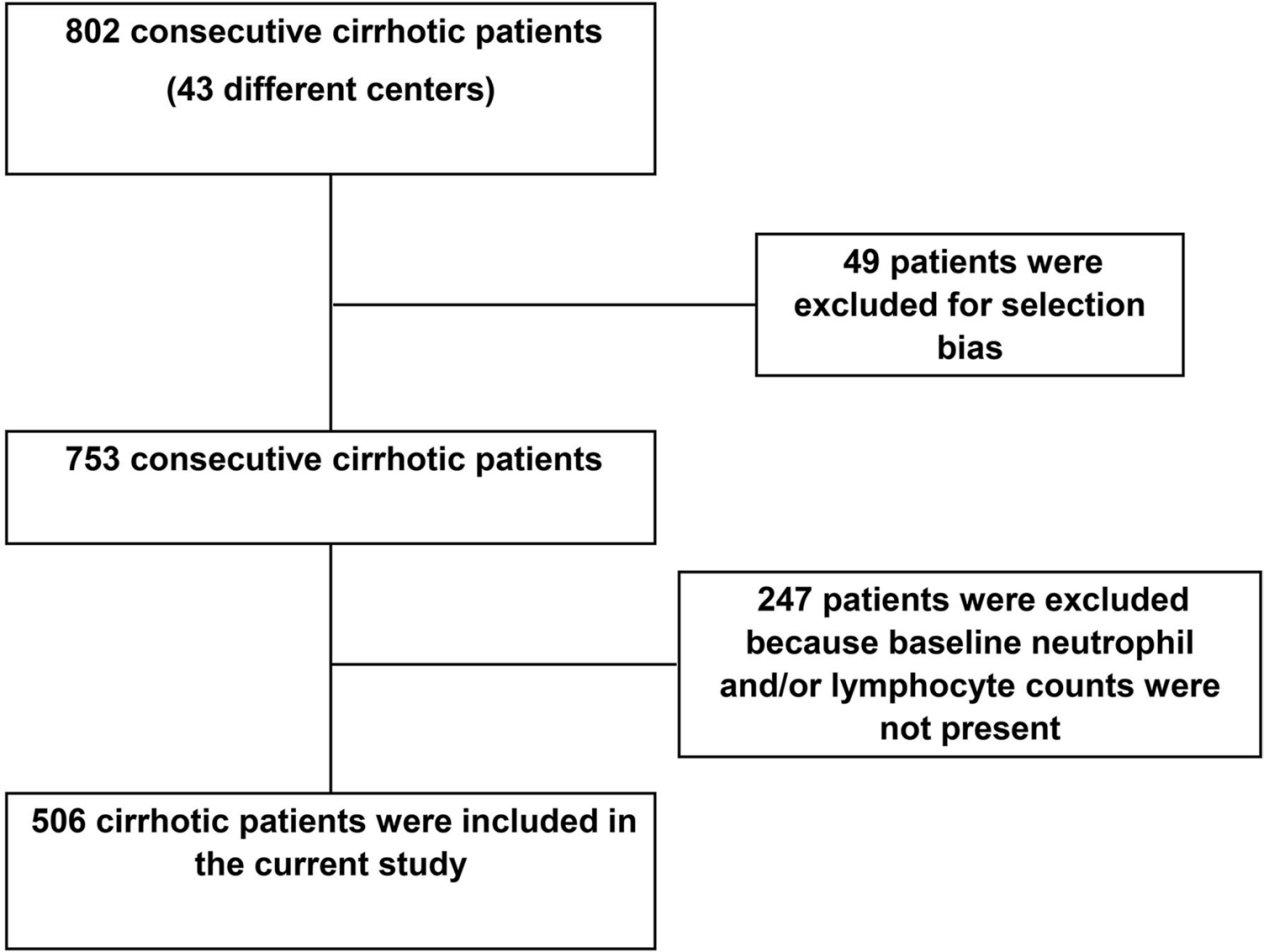
Flowchart of the study

Among the overall PRO-LIVER cohort (*n* = 753) [9] data on the neutrophil and lymphocyte counts were available in 506 patients (31.2% female sex; mean age: 64.8 ± 11.9 years), that were included in this analysis (Fig. 1).

The median values of NLR were 2.42 (IQR: 1.61–3.52). Baseline characteristics of the cohort included and stratified according to the median values of NLR are reported in Table 1. Overall, the predominant etiologies for LC were viral and alcoholic (44% and 27%, respectively); around half of the patients exhibited a mild disease severity with a (49%) Child–Pugh (CP) Class A, while CP classes B and C patients at baseline were 37% and 13%, respectively. Thus, most of the participants had no signs of ascites (56%) or encephalopathy (84%). An HCC was documented in 23% of cases. A baseline ultrasound-based diagnosis of PVT was present in 19% of patients, of whom only 13% were treated with any anticoagulant.

**Table 1:** Baseline clinical characteristics of patients with NLR levels above or below the median values

Variables	All patients	Patients with NLR < 2.42	Patients with NLR ≥ 2.42	*P*
N. of patients	506	253	253	
Age, years	64.8 ± 11.9	64.7 ± 11.6	65.0 ± 12.4	0.721
Male sex, *n* (%)	348 (69)	171 (68)	177 (70)	0.565
Etiology, *n* (%)				0.012
ALD, *n* (%)	138 (27)	52 (21)	86 (34)	
Viral, *n* (%)	221 (44)	120 (47)	101 (40)	
Autoimmune, *n* (%)	11 (2)	4 (2)	7 (3)	
NASH, *n* (%)	25 (5)	14 (5)	11 (4)	
Mixed, *n* (%)	58 (12)	36 (14)	22 (9)	
Others/unknown, *n* (%)	53 (10)	27 (11)	26 (10)	
Child–Pugh class				< 0.001
Class A, *n* (%)	246 (49)	159 (59)	96 (38)	
Class B, *n* (%)	186 (37)	85 (34)	101 (40)	
Class C, *n* (%)	74 (15)	18 (7)	56 (22)	
MELD score	10 [8–14]	9 [7–12]	12 [9–15]	< 0.001
HCC, *n* (%)	116 (23)	53 (21)	63 (25)	0.290
PVT, *n* (%)	94 (19)	35 (14)	59 (23)	0.006
Bilirubin, (mg/dl)	1.3 [0.8–2.3]	1.1 [0.8–1.9]	1.4 [0.9–2.8]	< 0.001
PT-INR	1.30 ± 0.33	1.26 ± 0.30	1.34 ± 0.35	0.003
Serum creatinine (mg/dl)	0.8 [0.7 −1.0]	0.8 [0.7–1.0]	0.9 [0.7–1.1]	0.072
Platelet count (× 10^3^/µl)	96 [68–139]	95 [67–138]	98 [69–144]	0.639
Serum albumin (g/l)	3.43 ± 0.61	3.49 ± 0.57	3.25 ± 0.67	< 0.001
Ascites				< 0.001
Absent, *n* (%)	286 (56)	176 (70)	110 (43)	
Responsive to diuretic therapy, *n* (%)	156 (31)	59 (23)	97 (38)	
Refractory, *n* (%)	64(13)	18 (7)	46 (18)	
Encephalopathy				< 0.001
Absent, *n* (%)	423 (84)	228 (90)	195 (77)	
Mild, *n* (%)	71 (14)	22 (9)	49 (19)	
Moderate to severe, *n* (%)	12 (2)	3 (1)	9 (4)	
Anticoagulants, *n* (%)	66 (13)	31 (12)	35 (14)	0.597
Warfarin, *n* (%)	18 (4)	12 (5)	6 (2)	0.232
LMWH, *n* (%)	39 (8)	16 (6)	23 (9)	
Fondaparinux, *n* (%)	9 (2)	3 (1)	46 (2)	

*ALD* alcohol-associated liver disease; *HCC* hepatocellular carcinoma; *LMWH* low-molecular-weight heparin; *MELD* Model for End-Stage Liver Disease; *NASH* nonalcoholic steatohepatitis; *PT-INR* prothrombin time-international normalized ratio; *PVT* portal vein thrombosis. Data are expressed as mean ± standard deviation, or median [interquartile range] or number (percentage)

Patients with NLR ≥ 2.42 were more likely to have an alcoholic etiology. They had a more advanced disease as documented by more frequent presence of encephalopathy and ascites, as well as higher levels of bilirubin, lower serum albumin and PT-INR prolongation, resulting in higher CP and Model for End-stage Liver Disease (MELD) scores (Table 1). Furthermore, they were more likely to have PVT at baseline.

### Variables associated with NLR levels above the median values.

Univariable logistic regression analyses confirmed that higher CP class (Class B vs. A, OR: 1.86, 95% CI: 1.23–2.73; *p* = 0.014; Class C vs. A, OR: 4.86, 95% CI 2.70–8.77; *p* < 0.001); alcoholic etiology (OR: 1.99; 95% CI: 1.3–2.97; *p* < 0.001); increasing MELD score (OR: 1.12; 95% CI: 1.07–1.17; *p* < 0.001); PVT at baseline (OR: 1.89; 95% CI: 1.19–3.00; *p* = 0.007); increased levels of bilirubin (OR: 1.26; 95% CI: 1.13–1.42; *p* < 0.001); increasing INR values (OR 2.38; 95% CI: 1.33–4.31; *p* = 0.004); increasing levels of serum creatinine (OR: 1.80, 95% CI: 1.13–2.87; *p* = 0.013); decreasing serum albumin levels (OR: 0.54; 95% CI: 0.41–0.73; *p* < 0.001); the presence at baseline of ascites (OR: 2.97; 95% CI: 2.06–4.28; *p* < 0.001); and encephalopathy (OR: 2.71; 95% CI: 1.63–4.50; *p* < 0.001) were associated with NLR ≥ 2.42.

A multiple logistic regression analysis showed that only higher CP class (Class B vs. A, OR: 1.64, 95% CI: 1.10–2.44; *p* = 0.014; Class C vs. A, OR: 4.19, 95% CI 2.29–7.64; *p* < 0.001), alcoholic etiology (OR: 1.61; 95% CI: 1.05–2.44; *p* = 0.027), and PVT at baseline (OR: 1.68; 95% CI: 1.04–2.71; *p* = 0.034) were independently associated with an increased likelihood of presenting with NLR above the median values.

### Survival analysis

**Fig. 2:**
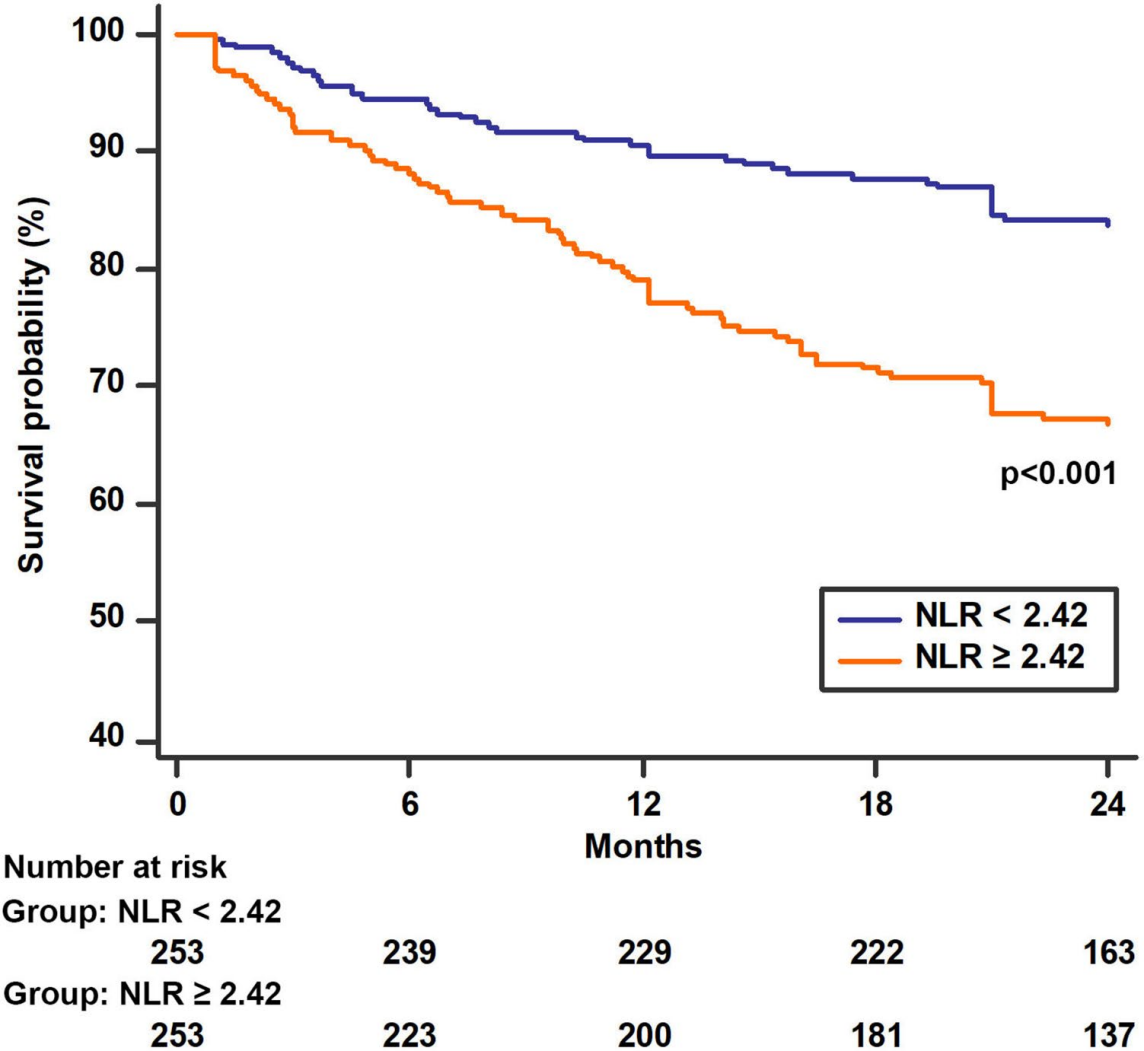
Kaplan–Meier estimates of time to mortality according to baseline NLR levels during the long-term follow-up

Patients were followed-up for a median of 21 (IQR: 7–24) months, yielding 690 patient-years of observation. During the observational period, 129 patients died, of whom 44 (17%) in the group with basal NLR < 2.42 and 85 (34%) in the one with basal NLR ≥ 2.42. Kaplan Meier curves showed a lower survival probability among patients with higher NLR values (Log-Rank *p* < 0.001, Fig. 2).

At univariable Cox regression analysis, NLR ≥ 2.42, age, Child–Pugh classes, MELD score, the presence of ascites, encephalopathy, HCC, PVT, lower baseline values of serum albumin, higher values of bilirubin and INR were all associated with mortality during the follow-up (Table 2).

**Table 2:** Clinical and laboratory characteristics associated with mortality: univariate analyses

Variables	HR	95% CI		*p*
Age, years	1.03	1.01	1.04	< 0.001
Male sex, *n* (%)	1.01	0.70	1.46	0.970
Etiology				
ALD	1.38	0.92	1.93	0.123
Viral	1.04	0.87	1.24	0.665
Autoimmune	0.89	0.56	1.42	0.636
NASH	0.98	0.81	1.19	0.852
Mixed	0.92	0.80	1.05	0.218
Child–Pugh class				
Class B vs. A	4.55	2.87	7.21	< 0.001
Class C vs. A	7.70	4.68	12.65	< 0.001
MELD score	1.11	1.08	1.13	< 0.001
HCC, *n* (%)	2.57	1.80	3.68	< 0.001
PVT, *n* (%)	3.09	2.13	4.48	< 0.001
NLR ≥ 2.42	2.15	1.49	3.10	< 0.001
Bilirubin, (mg/dL)	1.12	1.08	1.15	< 0.001
PT-INR	2.49	1.69	3.68	< 0.001
Serum creatinine (mg/dL)	1.25	1.10	1.42	< 0.001
Platelet count (× 10^3^/µL)	0.99	0.99	1.00	0.306
Serum albumin (g/L)	0.26	0.20	0.35	< 0.001
Ascites	4.10	2.80	6.00	< 0.001
Encephalopathy	2.13	1.43	3.16	< 0.001
Anticoagulants	1.27	0.77	2.09	0.348

*ALD* alcohol-associated liver disease; *HCC* hepatocellular carcinoma; *MELD* Model for End-Stage Liver Disease; *NASH* nonalcoholic steatohepatitis; *NLR* Neutrophil/Lymphocyte Ratio; *PT-INR* prothrombin time-international normalized ratio; *PVT* portal vein thrombosis

At multiple-adjusted Cox regression analysis, NLR ≥ 2.42 was associated with an increased hazard ratio (HR) of all-cause mortality (HR: 1.65, 95%CI: 1.18–2.44, *p* = 0.012), along with age, higher Child–Pugh classes, PVT and HCC (Table 3, Model A). NLR ≥ 2.42 was still associated with all-cause mortality when using MELD score instead of the Child–Pugh classification (HR: 1.62, 95%CI: 1.07–2.45, *p* = 0.013; Table 3, Model B).

**Table 3:** Multivariate COX regression analyses

Variables	HR	95% CI		*P*
MODEL A				
Age	1.024	1.008	1.041	0.003
CP Class B vs. A	3.610	2.258	5.772	< 0.001
CP Class C vs. A	6.599	3.896	11.177	< 0.001
HCC	2.110	1.451	3.067	< 0.001
PVT	1.823	1.230	2.700	0.003
NLR ≥ 2.42	1.651	1.118	2.438	0.012
MODEL B				
Age	1.019	1.002	1.035	0.026
HCC	1.903	1.301	2.784	< 0.001
PVT	2.001	1.351	2.965	< 0.001
NLR ≥ 2.42	1.619	1.070	2.449	0.023
MELD score	1.057	1.026	1.090	< 0.001
Ascites	2.493	1.614	3.850	< 0.001

Model A: using Child–Pugh classes, after adjusting for creatinine. Model B: using MELD score, after adjusting for encephalopathy

*CP* Child–Pugh; *HCC* hepatocellular carcinoma; *MELD* Model for End-Stage Liver Disease; *NASH* nonalcoholic steatohepatitis; *NLR* Neutrophil/Lymphocyte Ratio; *PVT* portal vein thrombosis

## Discussion

In this analysis from an unselected cohort of patients with LC of mixed etiologies, main findings are as follows: (1) a significant proportion of patients with LC has high levels of NLR, when compared to average values in the general population [4], (2) patients with high levels of NLR are more likely to be clinically more complex and with a more advanced stage of disease, supporting the role of inflammation as an additional factor promoting progression of liver damage; (3) high levels of NLR are independently associated with a significant higher risk of mortality, after adjustment for other potential confounders.

The NLR is a non-invasive cytological marker that reflects systemic inflammation. Its components, the total number of circulating neutrophils and lymphocytes, can be easily obtained in clinical practice through routine blood draws. Elevated NLR is associated with poor outcomes across various clinical conditions [5, 6, 11]. Our findings are consistent with existing literature, which documented an increased in NLR and the role of systemic inflammation in the pathogenesis of advanced liver diseases, including LC.

Systemic inflammation is a well-established factor in the pathogenesis of advanced-stage LC [12–14], which explains the common elevation of inflammatory markers like IL-6 in these patients [15]. Recent studies support the emerging concept that hepatocyte death, accompanied by the release of extracellular danger signals and activation of sterile inflammatory pathways, can trigger an intrahepatic, self-perpetuating harmful loop that is central to the development of liver fibrosis [16, 17]. The relationship between higher NLR levels and advanced inflammatory activity, as well as significant fibrosis, has been examined in patients with advanced liver damage from various causes. A cross-sectional study of 231 patients with liver biopsy-confirmed metabolic dysfunction-associated steatotic liver disease (MASLD) [18] found that NLR was significantly associated with histological features of MASLD, including steatosis, lobular inflammation, and fibrosis. While neutrophil infiltration in the liver exacerbates the progression of metabolic dysfunction-associated steatohepatitis (MASH), it also promotes the formation of new vascular channels, aiding in the repair of advanced liver injury [19].

In recent years, the potential of NLR to detect fibrosis has attracted interest also in chronic Hepatitis B Virus (HBV) and Hepatitis C Virus (HCV) infections. In HBV patients, the association between NLR and liver fibrosis has yielded mixed results. Some studies report a correlation between higher NLR values and more advanced fibrosis stages, while others find no significant association [20, 21]. In contrast, in chronic HCV patients, elevated NLR has been more consistently linked with advanced fibrosis and LC [6, 22]. However, data from Demet and colleagues [23] suggest no correlation between NLR and the degree of liver fibrosis, with no significant differences between cirrhotic and non-cirrhotic patients. These discrepancies likely arise from the differing nature of inflammatory responses and disease progression in HBV and HCV infections. Despite consistent findings across studies on the relationship between NLR and the severity of cirrhosis, significant variability in the NLR thresholds used to predict liver fibrosis or liver cirrhosis remains, making it challenging to establish standardized NLR cut-off value.[6, 24]

In the PRO-LIVER cohort, patients with elevated NLR levels were more likely to have a more advanced disease stage and greater clinical complexity. This finding is in line with different studies showing that neutrophil counts are higher and lymphocyte counts lower in patients with decompensated LC compared to those with compensated LC [20, 25], suggesting NLR as a predictive marker for decompensation in LC, particularly in advanced stages [20, 25].

Overall, variations in neutrophil and lymphocyte counts can reflect changes in the inflammatory state, both of which are significantly altered in cirrhotic patients. This may explain the high predictive value of NLR reported in the literature for infection episodes, acute decompensation, and mortality in cirrhotic patients. Of note, reflecting the new BAVENO VII definition of clinical compensation and recompensation in LC, it might be interesting to explore whether the NLR can identify different stages of the disease [26].

From a clinical point of view, the high risk of mortality for any cause is still a matter of immediate concern when managing patients with LC. Blood count is part of the routine lab testing in the follow-up of LC patients. The role of NLR as a predictor of mortality in patients with stable LC is consequently appealing to better stratify the probability of adverse outcomes. In our analyses, higher levels of NLR are independently associated with a significantly higher risk of mortality, after adjustment for other potential confounders. This finding is in line with several observational studies that consistently shown the association between higher NLR values and more advanced stages of LC, and with both short and long-term adverse outcomes [27, 28]. Most of these studies were conducted on cohorts of patients with an acute event leading to seek for medical care [27, 28]. Cai et al. found that in patients with decompensated LC, elevated NLR was linked to increased mortality at 6 months, 1 year, and 3 years, as well as to a higher risk of bacterial infections [7, 29]. Additionally, some evidence has shown that even after adjusting for MELD scores, higher NLR values predict an increased risk of mortality in patients awaiting liver transplantation [30].

However, literature largely lacks analyses of NLR as a predictor of mortality in patients with clinically stable LC. Biyik et al. [11] conducted a retrospective observational study on 145 patients with stable LC and found that the initial NLR value was significantly lower in surviving patients compared to non-surviving patients regardless of Child–Pugh or MELD scores. On the contrary, a prospective study by Kwon et al. [8] on 184 patients with LC further supported NLR's predictive value in patients with Child–Pugh class C, but not in those with Child–Pugh A or B.

In this light, our analysis confirmed and provided a novel contribution by demonstrating that NLR is independently associated with adverse outcomes even in a prospective, unselected cohort of patients with LC at different degrees of severity, reflecting real-world scenario. Specifically, patients with NLR ≥ 2.42 had an approximately 60% higher risk of mortality, independent from other significant known risk factors such as age, sex, PVT, HCC, and CP class at enrollment. While age, decompensated stage and complications such as PVT and HCC are well-known predictors of mortality [9, 31, 32], the prognostic role of NLR in an unselected LC cohort opens potential new scenarios for its application in clinical practice. High NLR might serve as an additional red flag to consider for a better management of high-risk patients.

Previous studies have proposed refining risk stratification using NLR [6]. Incorporating NLR into a more comprehensive score that includes both inflammatory and non-inflammatory causes of liver damage could improve the accuracy of prognostic risk stratification, particularly in advanced disease stages, in view of new evidence suggesting potential for partial regression of disease with new therapeutic approaches [33, 34].

Taken together, our results have clinical implications. First, the association observed between NLR and more advanced stages of LC aligns with the role of inflammation in disease progression; especially since we included LC of different etiologies. Second, the observation of higher risk of all-cause mortality in patients with NLR suggests that routine assessment of other biomarkers (beyond the liver-specific ones) may help in refine the risk stratification of these patients. This is also consistent with the observation that a significant proportion of deaths in LC patients result from non-liver-related causes [35], highlighting the need for closer surveillance of extrahepatic complications. In this scenario, NLR may offer a practical, readily available and cost-effective tool for assessing inflammation, and, although it cannot comprehensively evaluate inflammatory status, it may help identify those patients for whom a more in-depth workup may be needed. While further studies are needed to identify the optimal cut-off for this biomarker (and to evaluate whether a single cut-off may provide sufficient accuracy in all patients with LC, regardless of the etiology and grade of disease) our findings expand our knowledge on the potential role of NLR in LC and may provide basis for future research.

### Strengths and limitations

This analysis based on a real-world, nationwide multicenter cohort of Italian patients with LC, is of clinical relevance as representative of the complexity and heterogeneity of patients with advanced liver disease. Therefore, the results of this study may be translated to other similar Mediterranean cohorts.

Nevertheless, caution is needed in interpreting the results due to several limitations. Although we included more than 500 patients, larger cohorts of patients are needed to corroborate our findings. Patients were recruited from 43 Italian centers, but the ethnicity data were not recorded, limiting the generalizability of the results. We stratified our cohort according to the median values of NLR; nonetheless, our study is unable to provide evidence on the optimal cut-off of NLR to be applied to the general population of patients with LC, and therefore, caution should be applied when translating our results in clinical practice. Moreover, we used baseline data to calculate NLR; as inflammation is a dynamic process, we cannot exclude the contribution of any acute state in determining the values of neutrophil and lymphocyte. Finally, although we provided multiple adjusted regression models for the most important clinical characteristics (including disease stage), we cannot exclude the contribution of other unaccounted confounders.

## Conclusions

In this analysis from a multicenter Italian cohort of patients with LC, NLR values were independently associated with risk of 2-year all-cause mortality. We envision an implementation of current risk stratification and easy monitoring of adverse clinical outcomes incorporating NLR as a global marker of the complex immune balance in LC. Further studies are needed to confirm these findings.

## Data Availability

The datasets generated during and/or analysed during the current study are available from the corresponding author on reasonable request.
